# *EGFR* , *TP53* , and *CUL3* Triple Mutation in Non‐Small Cell Lung Cancer and its Potentially Poor Prognosis: A Case Report and Database Analysis

**DOI:** 10.1111/1759-7714.15523

**Published:** 2024-12-27

**Authors:** Hiroto Hatano, Tatsuya Yoshida, Ryoko Higashiyama, Masahiro Torasawa, Yuji Uehara, Yuichiro Ohe

**Affiliations:** ^1^ Department of Thoracic Oncology National Cancer Center Hospital Tokyo Japan; ^2^ Department of Respiratory Medicine National Center for Global Health and Medicine Tokyo Japan; ^3^ Department of Experimental Therapeutics National Cancer Center Hospital Tokyo Japan

**Keywords:** database, EGFR genes, KEAP1 protein, non‐small‐cell lung cancer, TP53 genes

## Abstract

Concurrent mutations in tumor protein p53 (TP53) or Kelch‐like ECH‐associated protein 1‐nuclear factor erythroid 2‐related factor 2‐pathway components are linked to poor outcomes in *epidermal growth factor receptor (EGFR*)‐mutant non‐small cell lung cancer (NSCLC), but the impact of triple mutations remains unclear. We report a case of *EGFR*‐, *TP53*‐, and *Cullin 3* (*CUL3*)‐mutant NSCLC in a 43‐year‐old woman with widespread metastases at diagnosis, including those in the contralateral lung, distant lymph nodes, pericardium, liver, bones, left adrenal gland, and brain. She received osimertinib as first‐line therapy, but pericardial effusion and liver metastases progressed rapidly over 3 months, and she was switched to carboplatin and pemetrexed. By the eighth cycle of pemetrexed, the bone metastases had progressed, resulting in disseminated intravascular coagulation (DIC) due to bone marrow carcinomatosis. The patient received third‐line therapy with albumin‐bound paclitaxel and fourth‐line therapy with docetaxel, but further treatment was suspended owing to DIC progression. She passed away 23 months after the initiation of osimertinib. Public database analysis revealed that the *EGFR*/*TP53*/*CUL3* triple mutation accounts for 0.4% of *EGFR*‐mutant NSCLC cases, yielding significantly shorter survival than *EGFR* mutations alone and likely shorter than *EGFR*/*TP53* double mutations. Gaining a deeper understanding of the clinical significance of coexisting genetic mutations in patients with *EGFR*‐mutant NSCLC will be crucial to develop future therapies.

## Graphical abstract

We report on a rare case with *EGFR*/*TP53*/*CUL3* triple mutations, with the rapid development of widespread metastases and bone marrow carcinomatosis despite the small size of the primary lesion at baseline. Additionally, public database analysis demonstrated the rarity of this triple mutation and its potentially poor prognosis among patients with *EGFR*‐mutant NSCLC.

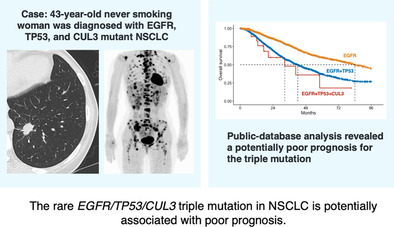

## 1 Introduction

Mutations of *epidermal growth factor receptor* (*EGFR*) are the primary therapeutic targets in non‐small cell lung cancer (NSCLC). For patients with stage IV NSCLC with *EGFR* mutations, EGFR‐tyrosine kinase inhibitors (EGFR‐TKIs) are the standard treatment. However, some patients exhibit primary resistance, and nearly all eventually become resistant [1].

Coexisting genetic mutations diminish the efficacy of EGFR‐TKI therapy. For instance, *tumor protein p53* (*TP53*) mutations, which are the most frequently coexisting mutations in patients with *EGFR*‐mutant NSCLC, are associated with a poorer prognosis [2]. Co‐mutations within the Kelch‐like ECH‐associated protein 1 (KEAP1)‐nuclear factor erythroid 2‐related factor 2 (NRF2) pathway, such as those in *KEAP1*, *nuclear factor erythroid‐derived 2‐like 2* (*NFE2L2*), and *Cullin 3* (*CUL3*), have also been reported to contribute to chemotherapy resistance and enhanced cell proliferation, worsening the prognosis of NSCLC [3]. Concomitant mutations of *TP53* and genes in the KEAP1‐NRF2 pathway in patients with *EGFR*‐mutant NSCLC are rare, and their clinical significance is unknown.

Here, we present a case of NSCLC with an *EGFR*/*TP53*/*CUL3* triple mutation. Additionally, we used a public database to assess the impact of the triple mutation on the prognosis of such patients.

## 2 Case Report

**FIGURE 1:**
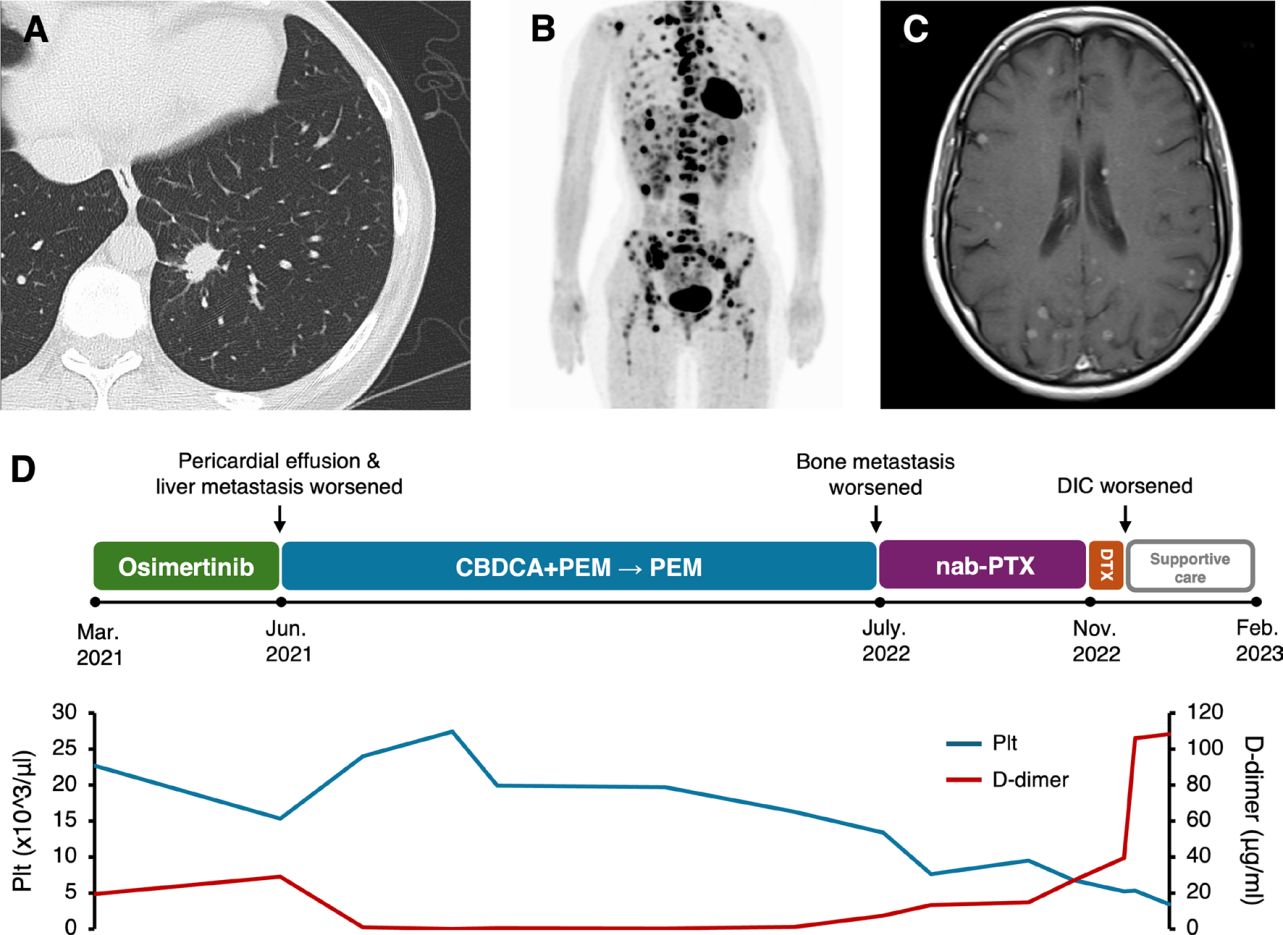
Clinical data of NSCLC case with EGFR/TP53/CUL3 triple mutation. (A) Chest computed tomography (CT) revealed a 16 mm × 11 mm nodule in the left lower lung lobe. (B) Positron‐emission tomography‐CT revealed liver metastasis, bone metastasis, and left adrenal metastasis. (C) Brain magnetic resonance imaging revealed multiple brain metastases. (D) Visual summary of the patient's clinical course. NSCLC, non‐small cell lung cancer, *EGFR*: *Epidermal growth factor receptor*, *TP53*: *Tumor protein p53*, *CUL3*: *Cullin 3*, DIC: Disseminated intravascular coagulation, CBDCA: Carboplatin, PEM: Pemetrexed, DTX: Docetaxel, Plt: Platelet.

A pulmonary nodule was discovered during a routine check‐up of a never‐smoking, 43‐year‐old woman with no history of medical conditions. Chest computed tomography (CT) revealed a nodule (Figure 1A) in the left lower lung lobe, multiple pulmonary metastases, enlargement of mediastinal lymph nodes, and pericardial effusion. Positron‐emission tomography‐CT revealed liver metastasis, large numbers of bone metastases, and left adrenal metastasis (Figure 1B), and brain magnetic resonance imaging revealed multiple brain metastases (Figure 1C). The patient was diagnosed with *EGFR*‐mutant lung adenocarcinoma. Comprehensive genome profiling revealed *EGFR* exon 19 deletion, *TP53*, and *CUL3* mutations (FoundationOne Liquid CDx assay; Foundation Medicine Inc., Cambridge, MA).

We started the patient on osimertinib. Three months later, her pulmonary lesions, mediastinal lymph node metastases, and brain metastases had reduced in size, but the pericardial effusion and liver metastases had progressed rapidly. Thus, her treatment was switched to carboplatin (CBDCA) and pemetrexed (PEM). Four cycles of CBDCA + PEM reduced the pericardial effusion and liver metastases. However, by the eighth cycle of PEM, the bone metastases had progressed, resulting in disseminated intravascular coagulation (DIC) due to bone marrow carcinomatosis (platelet count: 6.0 × 10^3^]/μL, D‐dimer: 29.3 μg/mL, and prothrombin time‐international normalized ratio: 1.29). Thus, the patient was initiated on third‐line therapy with albumin‐bound paclitaxel, and fourth‐line therapy with docetaxel, but further treatment was suspended owing to DIC progression. She passed away 23 months after the initiation of osimertinib (Figure 1D).

### 2.1 Database Analysis

**FIGURE 2:**
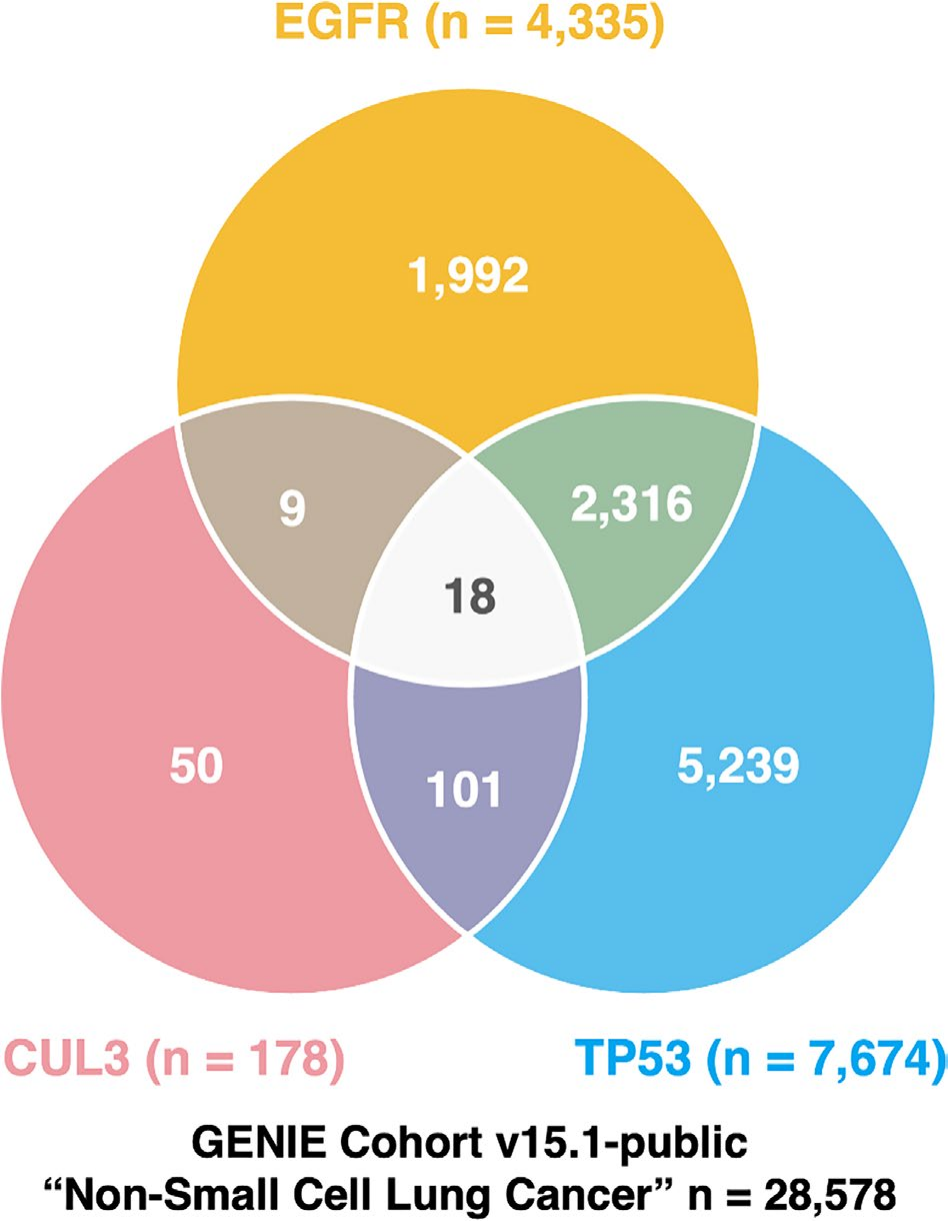
Frequencies of EGFR, TP53, and CUL3 mutations among patients with non‐small cell carcinoma in the GENIE cohort v15.1‐public database. Among patients with *EGFR* mutations, 1992 had an *EGFR* mutation without *TP53* or *CUL3* mutations, 2316 had *EGFR*/*TP53* double mutations, and 18 had *EGFR*/*TP53*/*CUL3* triple mutations. *EGFR*: *Epidermal growth factor receptor*, *TP53*: *Tumor protein p53*, *CUL3*: *Cullin 3*, GENIE: Genomics, Evidence, Neoplasia, Information, Exchange.

In light of this case, we investigated the frequency of such triple mutations and their impact on the prognosis of patients with *EGFR*‐mutant NSCLC (Supplementary Methods). In the Genomics, Evidence, Neoplasia, Information, Exchange Cohort v15.1‐public database [4], 4335 patients with NSCLC had *EGFR* mutations, 7674 had *TP53* mutations, and 178 had *CUL3* mutations. Overall, 1992 had only *EGFR* mutations (46.0%), 2316 had *EGFR*/*TP53* double mutations (53.4%), and 18 had *EGFR*/*TP53*/*CUL3* triple mutations (0.4%) (Figure 2). No differences in the accessible baseline characteristics were observed (Supplementary Table 1). Additionally, the frequency of *CUL3* mutations co‐occurring with simultaneous *EGFR* and *TP53* mutations was lower than that of *KEAP1* mutations and as low as that of *NFE2L2* mutations (18 [0.8%] vs. 73 [3.1%] vs. 15 [0.6%]).

**FIGURE 3:**
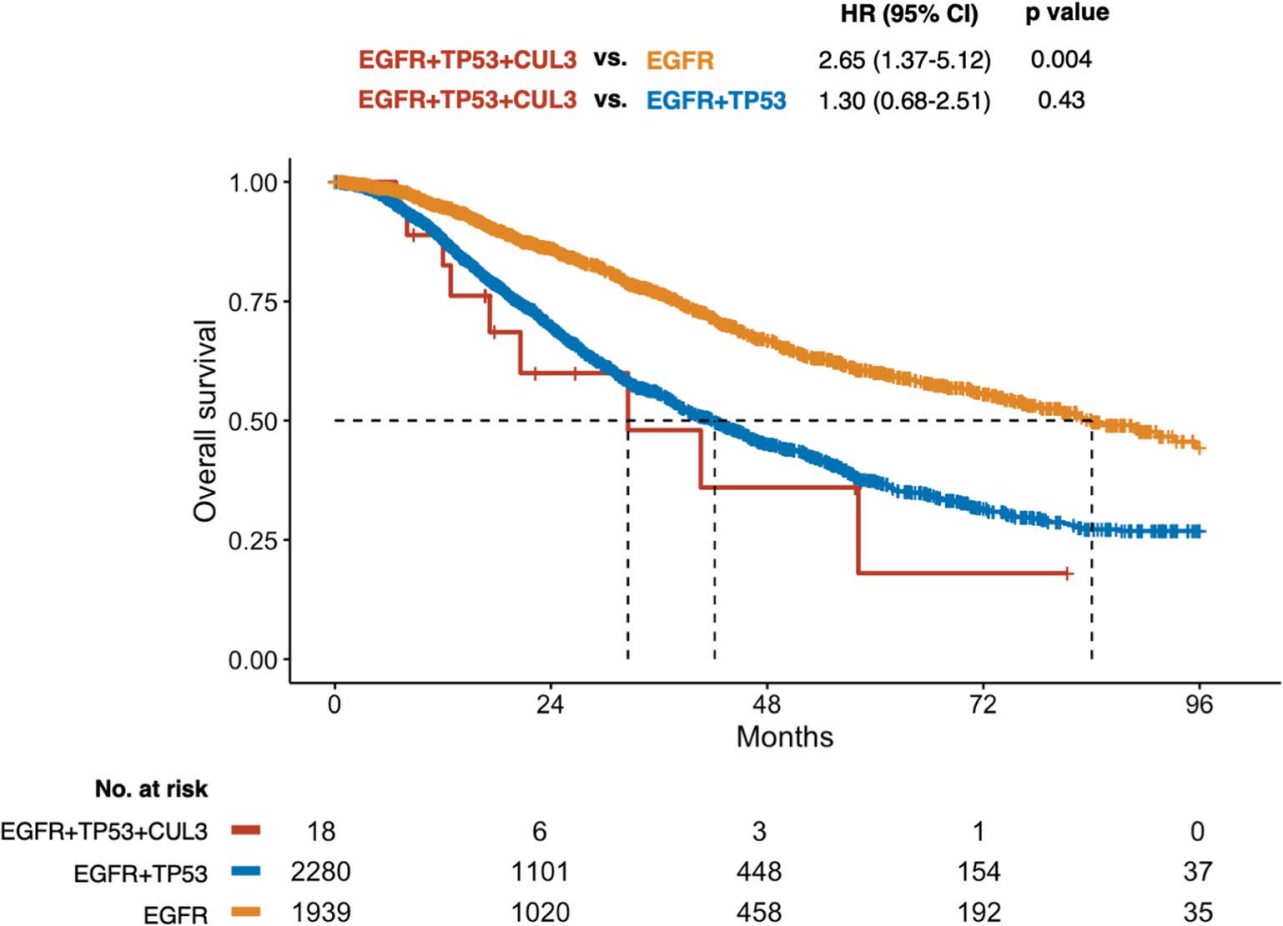
Kaplan–Meier curves of overall survival, comparing EGFR, EGFR/TP53, and EGFR/TP53/CUL3 mutations. The *EGFR*/*TP53*/*CUL3* triple mutation yielded a worse prognosis than the *EGFR* single mutation (HR: 2.65, 95% CI: 1.37–5.12, *p* = 0.004) and appeared to yield a worse prognosis than the *EGFR*/*TP53* double mutation (HR: 1.30, 95% CI: 0.68–2.51), although the difference was not statistically significant (*p* = 0.43). The median OS was 84.0 (*EGFR*), 42.2 (*EGFR*/*TP53*), and 32.6 (*EGFR*/*TP53*/*CUL3*) months. *EGFR*: *Epidermal growth factor receptor*, *TP53*: *Tumor protein p53*, *CUL3*: *Cullin 3*, HR: Hazard ratio, 95% CI: 95% confidence interval, OS: Overall survival.

The *EGFR*/*TP53*/*CUL3* triple mutation yielded a worse prognosis than the *EGFR* single mutation, with a hazard ratio (HR) of 2.65 (95% confidence interval [CI]: 1.37–5.12, *p* = 0.004). It also seemed to yield a worse prognosis than the *EGFR*/*TP53* double mutation (HR: 1.30, 95% CI: 0.68–2.51), although the difference was not significant (*p* = 0.43) (Figure 3). The median overall survival (OS) was 84.0 (*EGFR*), 42.2 (*EGFR*/*TP53*), and 32.6 (*EGFR*/*TP53*/*CUL3*) months.

## 3 Discussion

Here, we reported on a rare case with *EGFR*/*TP53*/*CUL3* triple mutations, with the rapid development of widespread metastases and bone marrow carcinomatosis despite the small size of the primary lesion at baseline. Furthermore, public database analysis demonstrated the rarity of this triple mutation and its potentially poor prognosis among patients with *EGFR*‐mutant NSCLC.

Among mutations co‐occurring with *EGFR* mutations, those in *TP53* are the most common, constituting 54.6%–64.5% of *EGFR*‐mutant cases [5]. Mutations in the KEAP1‐NRF2 pathway are relatively rare, accounting for 7% of patients with *EGFR*‐mutant lung adenocarcinoma in one study [3]. In *Kirsten rat sarcoma viral oncogene homolog*‐mutant lung adenocarcinoma, *TP53* and *KEAP1* mutations tend to occur independently, forming biologically distinct subgroups [6]. However, the frequency of co‐occurring *TP53* and KEAP1‐NRF2‐pathway mutations in patients with *EGFR*‐mutant NSCLC, as well as their impact on the prognosis, remains unknown.

The p53 protein, encoded by the *TP53* gene, regulates the cell cycle, promotes DNA repair, and induces apoptosis when DNA damage accumulates, thereby preventing cancer development. *TP53* mutations can lead to enhanced cancer‐cell proliferation, increased metastasis and invasion, and the development of drug resistance [7]. Co‐mutation of *TP53* in *EGFR*‐mutant NSCLC is reportedly associated with reduced responsiveness to first‐ or second‐generation EGFR‐TKI treatment (progression‐free survival [PFS]: 6.5 vs. 14 months, *p* = 0.025; OS: 28 vs. 52 months, *p* = 0.023) [2]. *TP53* co‐mutations are reported as poor prognostic factors even when osimertinib, a third‐generation EGFR‐TKI, is used in the second‐line setting or later for patients with *EGFR* T790M mutations [8].

*CUL3* is a component of the KEAP1‐NRF2 pathway. NRF2 controls the expression of antioxidants and is regulated by ubiquitination and degradation via CUL3 E3 ligases. KEAP1 facilitates NRF2 ubiquitination, via CUL3, and subsequent NRF2 degradation [9]. Mutations in these genes can result in sustained activation of NRF2, enhancing cancer‐cell proliferation, metastasis, and chemotherapy resistance [10]. In one study, concomitant mutations in the KEAP1‐NRF2 pathway in *EGFR*‐mutant NSCLC were associated with a shorter PFS among patients who were mostly treated with first‐ and second‐generation EGFR‐TKIs (4.7 vs. 13.0 months, *p* = 0.0014), but most of those mutations occurred in *KEAP1* or *NFE2L2* [3]. Clinical data on osimertinib remain limited, but an in vivo study revealed that KEAP1 deficiency reduces the efficacy of osimertinib in *EGFR*/*TP53* double‐mutant lung cancer [11]. *CUL3* mutations are less common than those in other KEAP1‐NRF2‐pathway components, and the effect of triple mutations including *CUL3* on clinical outcomes of patients with *EGFR*‐mutant NSCLC is unclear.

Our database analysis revealed that *EGFR*/*TP53*/*CUL3* triple mutations account for only 0.4% of *EGFR*‐mutant NSCLC cases and seemed associated with a lower OS than *EGFR*/*TP53* double mutations, although not significantly so (possibly owing to the small sample size). Furthermore, our patient exhibited rapid progression and severe complications, including bone marrow carcinomatosis, despite the primary lesion at baseline being small. *EGFR*‐mutant lung adenocarcinoma with coexisting *TP53* mutations is more prone to exhibit whole‐genome doubling than *EGFR* mutations alone, often leading to copy‐number alterations and an increased likelihood of a mixed response [12]. Abnormalities in the KEAP1‐NRF2 pathway are also associated with genomic instability due to impaired regulation of oxidative stress [13]. The addition of *CUL3* mutations to *EGFR*/*TP53* double mutations may further complicate resistance mechanisms, potentially resulting in unique clinical outcomes. Furthermore, a reduction in reactive oxygen species (ROS) is a potential mechanism of acquired resistance to osimertinib [14]. *CUL3* mutations are likely to contribute to osimertinib resistance by decreasing ROS levels through the accumulation of NRF2.

These previous studies, along with our data, suggest that tailored treatment strategies based on co‐mutation status may be effective for *EGFR*‐mutant NSCLC. Amivantamab plus lazertinib prolongs PFS in *EGFR*‐mutant NSCLC with *TP53* mutations compared with osimertinib [15]. For KEAP1‐NRF2‐pathway mutations, the addition of ROS‐inducing treatment, such as chemotherapy or targeted therapies (e.g., nicotinamide adenine dinucleotide kinase phosphorylation inhibitors [14]), to EGFR‐TKI therapy may enhance treatment effectiveness. A deeper understanding of coexisting mutations associated with *EGFR* is crucial for optimal treatment selection and the development of new therapies, highlighting the need for the continued accumulation of cases.

We reported on a rare case of NSCLC with *EGFR*/*TP53*/*CUL3* triple mutations, highlighting the potential poor prognosis associated with such a combination of mutations. Gaining a deeper understanding of the clinical significance of coexisting genetic mutations in patients with *EGFR*‐mutant NSCLC is crucial for the development of future therapies.

## Author Contributions

**H.H.:** writing ‐ Original Draft. **T.Y., R.H., M.T., Y.U., and Y.O.:** writing ‐ Review and Editing. **T.Y.:** conceptualization. All the authors approved the final manuscript.

## Disclosure

T.Y. received grants and personal fees from Amgen, AstraZeneca, Bristol Myers Squibb, Chugai Pharmaceutical, Roche, Daiichi Sankyo, Novartis Pharmaceuticals, Ono Pharmaceutical, Takeda Pharmaceutical, and Eli Lilly; grants from AbbVie and Merck Sharp & Dohme; personal fees from Bayer, UCB, Boehringer Ingelheim, and Pfizer. Y.O. received grants and personal fees from AstraZeneca, Bristol Myers Squibb, Chugai Pharmaceutical, Eli Lilly, Novartis, Ono Pharmaceutical, Pfizer, Taiho Pharmaceutical, and Takeda Pharmaceutical; grants from Janssen Pharmaceuticals; personal fees from Amgen, Bayer, Boehringer Ingelheim, Eisai, Kyowa Kirin, and Nippon Kayaku. The remaining authors declare no competing interests.

## Consent

The patient provided informed consent for publication.

## Conflicts of Interest

The authors declare no conflicts of interest.

## Supporting information


**Data S1** Supporting Information

## Data Availability

The datasets used and/or analyzed during the current study are available from the corresponding author on reasonable request.
