# Supplementary material for: EGFR , TP53 , and CUL3 Triple Mutation in Non‐Small Cell Lung Cancer and its Potentially Poor Prognosis: A Case Report and Database Analysis
Source: Thorac Cancer. 2024 Dec 27;16(3):e15523. doi: 10.1111/1759-7714.15523 (PMC11788012; doi:10.1111/1759-7714.15523)
Supplement: Supplementary file 1 — Data S1 Supporting Information [file TCA-16-e15523-s001.docx]

**Supplementary Methods**

**Database analysis**

We accessed the AACR GENIE Cohort v15.1-public data on cBioPortal (https://www.cbioportal.org/) on July 4th, 2024. Patients registered as having “non-small cell lung cancer” in the cohort were examined, and individuals with mutations in *EGFR*, *TP53*, and *CUL3* were extracted. The number of patients with each mutation and coexisting mutations were counted.

Among patients with *EGFR* mutations, three groups were identified: those with only *EGFR* mutations, those with *EGFR* + *TP53* double mutations, and those with *EGFR* + *TP53* + *CUL3* triple mutations. The overall survival of these groups were compared. Survival status was determined according to the values in the “Vital Status” column in the database. For deceased patients, survival time was calculated as (“Interval in days from DOB to DOD” / 365.25 – “Age at Which Sequencing was Reported”) × 12. For surviving patients, follow-up time was calculated as (“Interval in days from DOB to date of last contact” / 365.25 – “Age at Which Sequencing was Reported”) × 12, with a maximum follow-up period of 96 months. Cases were excluded from the survival analysis if the “Age at Which Sequencing was Reported” was “NA,” “Unknown,” or “>89”; the “Interval in days from DOB to date of last contact” was “NA,” “<6570,” or “>32485”; the “Interval in days from DOB to DOD” was “<6570” or “>32485”; no “Vital Status” information was available; or the “Interval in days from DOB to date of last contact / 365.25” or “Interval in days from DOB to DOD / 365.25” value was less than the “Age at Which Sequencing was Reported.”

**Statistical analysis**

Survival analysis was conducted using Kaplan-Meier curves, and a Cox proportional-hazards model was used to calculate hazard ratios with 95% confidence intervals. All data processing and statistical analyses were performed using R software (R Foundation for Statistical Computing, Vienna, Austria). The R packages “dplyr,” “tidyr,” and “tidyverse” were used for data formatting, and “survival” and “survminer” were used for survival analysis and plot generation.

**Supplementary Table 1. Comparison of the baseline characteristics that were accessible on the GENIE database.**

| Variables | | EGFR (n=1,939) | EGFR + TP53 (n=2,280) | EGFR + TP53  + CUL3 (n=18) |
| --- | --- | --- | --- | --- |
| Age | | 67.4±10.2 | 64.3±10.8 | 68.8±8.3 |
| Sex | |  |  |  |
|  | Male | 581 (30.0) | 734 (32.2) | 8 (44.4) |
|  | Female | 1,358 (70.0) | 1,545 (67.8) | 10 (55.6) |
|  | Unknown | 0 (0) | 1 (0.0) | 0 (0) |
| Race | |  |  |  |
|  | White | 1,199 (61.8) | 1,469 (64.4) | 12 (66.7) |
|  | Black | 114 (5.9) | 149 (6.5) | 0 (0) |
|  | Asian | 380 (19.6) | 395 (17.3) | 5 (27.8) |
|  | Native American | 2 (0.1) | 3 (0.1) | 0 (0) |
|  | Pacific islander | 1 (0.1) | 3 (0.1) | 0 (0) |
|  | Others | 97 (5.0) | 109 (4.8) | 0 (0) |
|  | Unknown | 109 (5.6) | 152 (6.7) | 1 (5.5) |
